# Current state-of-the-art of adrenal surgery in Italy: the cancer risk in surgical adrenal lesions (CRISAL) survey

**DOI:** 10.1007/s13304-025-02139-8

**Published:** 2025-03-17

**Authors:** Diletta Corallino, Roberto Passera, Marco Inama, CRISAL (Cancer Risk In Surgical Adrenal Lesions) Collaborative Group, Diletta Corallino, Marco Inama, Francesca Abbatini, Stefano Agnesi, Ferdinando Agresta, Alberto Aiolfi, Laura Alberici, Giovanni Alemanno, Marco Ettore Allaix, Michele Ammendola, Pietro Maria Amodio, Marco Anania, Andrea Pisani Ceretti, Jacopo Andreuccetti, Roberta Angelico, Pierluigi Angelini, Mario Annecchiarico, Alfredo Annicchiarico, Pietro Anoldo, Amedeo Antonelli, Massimiliano Ardu, Giulio Argenio, Gabriela Aracelly Arroyo Murillo, Riccardo Avantifiori, Giulia Bagaglini, Gian Luca Baiocchi, Edoardo Baldini, Alberto Balduzzi, Francesco Balestra, Andrea Balla, Filippo Banchini, Elisa Bannone, Ilaria Benzoni, Lorenza Beomonte Zobel, Francesco Bianco, Arianna Birindelli, Cristina Bombardini, Luca Domenico Bonomo, Andrea Bottari, Marta Botti, Paolo Brazzarola, Francesco Brucchi, Simone Buccianti, Oreste Claudio Buonomo, Giacomo Calini, Roberto Cammarata, Tommaso Campagnaro, Sonia Cappelli, Marianna Capuano, Filippo Carannante, Gabriele Carbone, Luca Cardinali, Francesco Maria Carrano, Gianmaria Casoni Pattacini, Gianluca Cassese, Elisa Cassinotti, Antonio Castaldi, Fausto Catena, Giuseppe Cavallaro, Graziano Ceccarelli, Marta Celiento, Giovanni Cestaro, Vittorio Cherchi, Pasquale Cianci, Bruno Cirillo, Marco Clementi, Lucrezia Clocchiatti, Diego Coletta, Annalisa Comandatore, Luigi Eduardo Conte, Giovanni Conzo, Alessandro Coppola, Maurizio Costantini, Mihail Creciun, Diego Cuccurullo, Giuseppe Currò, Anna D’Amore, Maria Vittoria D’Addetta, Giorgio Dalmonte, Michele De Capua, Giuseppe Massimiliano De Luca, Maurizio De Luca, Nicolò De Manzini, Paolino De Marco, Belinda De Simone, Federico De Stefano, Sara Dedoni, Daniele Delogu, Annamaria De Bella, Giuseppe De Buono, Armando De Dato, Giacomo Di Filippo, Gregorio Di Franco, Nicola Di Lorenzo, Salomone Di Saverio, Andrea Divizia, Stefano D’Ugo, Ugo Elmore, Kevin Episodio, Emilio Eugeni, Giuseppe Evola, Nicolò Falco, Chiara Fantozzi, Alessia Fassari, Salvatore Fazzotta, Agostino Fernicola, Federico Festa, Irene Fiume, Tommaso Fontana, Edoardo Forcignanó, Gianluca Fornoni, Laura Fortuna, Alice Francescato, Marzia Franceschilli, Pietro Fransvea, Francesco Frattini, Giuseppe Frazzetta, Niccolò Furbetta, Raffaele Galleano, Giovanni Maria Garbarino, Enza Gelormini, Omar Ghazouani, Marco Giacometti, Alessio Giordano, Francesco Giovanardi, Giuseppe Giuliani, Ugo Giustizieri, Simone Guadagni, Tommaso Guagni, Anna Guariniello, Andrea Martina Guida, Giulio Iacob, Salvatore Incardona, Sara Ingallinella, Zoe Larghi Laureiro, Sara Lauricella, Leandro Siragusa, Silvana Leanza, Luca Lepre, Enrico Lodo, Sara Lucchese, Andrea Lucchi, Luigi Luzza, Andrea Pierre Luzzi, Carmen Maccagnano, Federico Maggi, Tommaso Maria Manzia, Sara Maritato, Nirvana Maroni, Riccardo Marsengo, Irene Marziali, Manuela Mastronardi, Marco Materazzo, Angela Maurizi, Gennaro Mazzarella, Francesca Meoli, David Merlini, Ilenia Merlini, Alessandra Micalizzi, Michail Vailas, Michele Minuto, Sarah Molfino, Serena Molica, Luca Morelli, Andrea Morini, Barbara Mullineris, Bruno Nardo, Giuseppe Navarra, Antonella Nicotera, Greta Olivari, Stefano Olmi, Monica Ortenzi, Paolo Ossola, Luca Ottaviani, Mario Pacilli, Alessandro M. Paganini, Livia Palmieri, Giuseppe Palomba, Vincenzo Papagni, Giulia Paradiso, Rocco Pasqua, Federico Passagnoli, Francesco Pata, Alberto Patriti, Giovanna Pavone, Domiziana Pedini, Fabio Pelle, Marco Pellicciaro, Vito Pende, Francesco Pennestrì, Bruno Perotti, Teresa Perra, Nicola Perrotta, Filippo Petrelli, Niccolò Petrucciani, Biagi Picardi, Andrea Picchetto, Stefania Piccioni, Chiara Piceni, Giulia Pietricola, Felice Pirozzi, Paolo Pizzini, Mauro Podda, Gaetano Poillucci, Alberto Porcu, Gianmario Edoardo Porcu, Priscilla Francesca Procopio, Lorenzo Provinciali, Francesco Puccetti, Ilaria Puccica, Eleonora Rapanotti, Antonia Rizzuto, Fabrizio Romano, Riccardo Rosati, Francesco Roscio, Leonardo Rossi, Stefano Rossi, Margherita Sandano, Federica Saraceno, Alberto Sartori, Paolina Saullo, Giovanni Scudo, Ardit Seitaj, Bruno Sensi, Marta Spalluto, Domenico Tamburrino, Mariarita Tarallo, Ernesto Tartaglia, Nicola Tartaglia, Giovanni Terrosu, Pier Luigi Tilocca, Flavio Tirelli, Luca Tirloni, Lorenza Trentavizi, Sofia Usai, Valeria Usai, Alessandro Ussia, Samuele Vaccari, Maria Rosaria Valenti, Gianluca Vanni, Samantha Vellei, Paolo Vincenzi, Antonio Vitiello, Mattia Zambon, Daniele Zigiotto, Maurizio Zizzo, Ugo Boggi Riccardo, Casadei Massimiliano, Fabozzi Mario, Guerrieri Gabriele, Materazzi Gianluigi, Moretto Micaela, Piccoli Paolo Prosperi, SICE (Italian Society of Endoscopic Surgery and New Technologies)

**Affiliations:** 1 Hepatobiliary Surgery Division, IRCCS San Raffaele Scientific Institute, Via Olgettina 60, 20132 Milan, Italy; 2 Department of General Surgery and Surgical Specialties, Sapienza University of Rome, Viale del Policlinico 155, 00161 Rome, Italy; 3 Nuclear Medicine, Department of Medical Sciences, AOU Città Della Salute E Della Scienza Di Torino, University of Turin, 10126 Turin, Italy; 4 General and Mininvasive Surgery Department, Pederzoli Hospital, Peschiera del Garda, Verona, Italy

**Keywords:** Adrenal lesions, Cancer risk, Expert adrenal surgeon, High-volume center, Surgery, Survey

## Abstract

Adrenalectomies are growing worldwide because of the frequent diagnosis of incidentaloma and the use of minimally invasive surgery (MIS). The factors used to identify a malignant lesion and the best surgical technique are uncertain. In this context, the definition of high-volume center and expert surgeon is under debate. The Italian Society of Endoscopic Surgery and New Technologies (SICE) developed a nationwide survey to investigate the state-of-the-art of adrenal surgery in Italy. A web-based survey comprising 37 questions was developed and distributed to Italian surgeons involved in adrenal surgery. Two hundred forty-eight answers were analyzed. Consensus was reached among the survey participants regarding local infiltration (83%) and rapid growth of the lesion (81%) as markers of malignancy. Nearly 30% of the participants used MIS in case of malignant adrenal lesions. The lateral (50%) and anterior transperitoneal (44%) approaches were the most common among Italian surgeons. Approximately 40% of participants believe that 20–40 adrenalectomies/year are needed to define an expert surgeon and at least 20 procedures/year to define a high-volume center. Approximately half of participants performed < 10 adrenalectomies/year in centers with a median volume < 10 procedures/year**.** Based on participant feedback, this survey highlights local infiltration and rapid growth as the most significant markers of malignant adrenal lesions. While open adrenalectomy remains the gold standard for suspected malignant lesions, nearly 30% of the participants practice MIS even in these cases. The lateral and anterior transperitoneal approaches emerge as the most familiar for Italian surgeons. A substantial proportion of Italian patients with adrenal lesions undergo surgery performed by surgeons with an annual case volume < 10 procedures, at centers with a low annual volume of adrenalectomies. Moreover, there is a lack of standardized definitions for ‘expert surgeon’ and ‘high-volume center’ in this context.

## Introduction

The global rate of adrenal surgery has increased steadily over the past 2 decades [1–4]. This trend can be attributed to the early diagnosis of incidentalomas and the more frequent use of minimally invasive surgery (MIS) [5–7].

The risk of adrenocortical carcinoma (ACC) rises with the increasing lesion size [8–11]. While hormonal activity is observed in up to 60% of malignant adrenal lesions [12–14] and hypercortisolism is a strong indicator of malignancy [15, 16], data on the specific cancer risk associated with secreting lesions remain unclear.

As reported in the literature, cancer risk is critical in choosing surgical approach [15, 16]. Although current guidelines suggest open adrenalectomy for lesions with preoperative features suspicious of malignancy (size ≥ 6 cm, radiological features suggestive of malignancy, history of neoplastic disease, rapid growth) [4, 8–10], several authors have reported the safety and feasibility of MIS in these cases too [1, 4, 5].

Since no clear superiority of one MIS procedure over another (lateral, posterior, anterior approach) in terms of perioperative outcomes has been demonstrated, the guidelines agree on using surgeon’s most familiar approach [8–10].

Finally, guidelines emphasize the importance of a multidisciplinary management and surgical expertise in the case of patients undergoing adrenalectomy [17]; however, in Italy, the fundamental requirements to define a high-volume center in adrenal surgery and an “experienced” adrenal pathology surgeon are still debated [3, 4].

This nationwide survey aims to report the current state-of-the-art of adrenal surgery in Italy. Specifically, the CRISAL survey explores the criteria currently used to define an oncological-risky lesion in adrenal gland, a high-volume center and experienced adrenal surgeons. Moreover, the survey reports the different surgical approaches and the application of Enhanced Recovery After Surgery (ERAS) pathway in adrenalectomy.

## Methods

This study was conducted according to the ethical guidelines for good research and practice published by the World Health Organization [18] and to the E-Surveys Checklist for Reporting Results of Internet (CHERRIES) [19].

The steering committee of the CRISAL study (D.C., M.I., and R.P) has lunched, under the aegis of The Italian Society of Endoscopic Surgery and New Technologies (SICE), a web-based survey to investigate the current state-of-the-art regarding adrenal surgery and the risk of cancer in surgical adrenal lesions in Italy.

The questionnaire was developed by the steering committee. Once a general agreement among the steering committee members concerning all questions was reached, the electronic questionnaire was tested for its functionality and published online using Google Form (Google LLC, Mountain View, California US). The link to complete the questionnaire was sent to all SICE members and other potential participants by web invitation.

The questionnaire consisted of 37 questions divided as follows: personal data (11 questions), personal opinions (10 questions), and data from clinical practice (16 questions). All answers were mandatory and the estimated mean time to complete the survey was 10 min.

The questionnaire was available online from March 15, 2023, to December 4, 2023. In addition, the link was sent through the mailing list of SICE and personal invitations from the steering committee. Moreover, the link was available on the SICE website (https://siceitalia.com/area-medico/studi-sperimentali/study-augmented-reality), in the area dedicated to the scientific research that is proposed and endorsed by the Society.

## Statistical analysis

Continuous covariates were reported as median and interquartile range (IQR), while categorical ones as absolute and relative frequencies. The Fisher’s exact test and the Mann–Whitney one were applied for the inferential analyses, for categorical and continuous variables, respectively. All *p* values were obtained by the two-sided exact method, at the conventional 5% significance level. Consensus was considered to have been reached when the percentage of agreement was ≥ 75%, as reported in the literature [20]. Data were analyzed as of June 2024 by R 4.4.0 (R Foundation for Statistical Computing, Vienna-A, http://www.R-project.org).

## Results

Two-hundred and forty-eight Italian surgeons sent their complete responses to the questionnaire, and their answers were analyzed. Tables 1–3 report the results of the participants’ data. Most of the participants were men (125, 68%) with a median age of 36 years (IQR: 32–45), and most of them were attending surgeons (201, 81%), while the others were residents in surgery (47, 19%).

**Table 1:** Results from personal data

Gender ratio, women (%):men (%)	76 (30.6):172 (69.4)
Median age, years (IQR)	36 (32–45)
Residents, *n* (%)	47 (19.0)
Clinical practice hospital, *n* (%) Academic Public Private	111 (44.8) 122 (49.2) 15 (6.0)
Italian Society of Endoscopic Surgery (SICE) member, *n* (%)	115 (46.4)
Median time of practice after the end of the residency, years (IQR)	4 (1–12)

*IQR* interquartile range

**Fig. 1:**
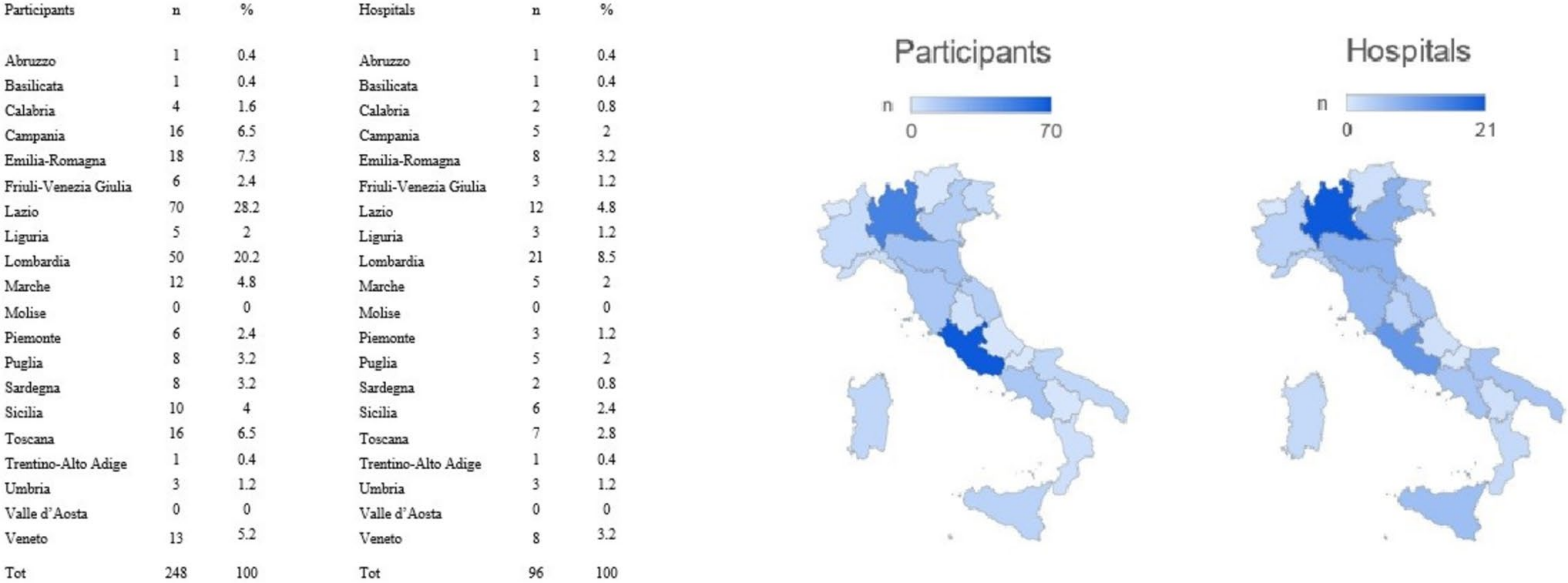
Participants’ Italian regions of practice

Most of the participants worked at a Hospital Agency integrated with the National Health System Hospital (122, 49%) or a university-affiliated agency (111, 45%) (Table 1). Only 6% of the participants practice in private hospitals. As reported in Fig. 1, many participants were from Northern and Central Italy, and 115 participants (46%) were SICE members.

Table 2 reports results on the participants’ personal opinions section. Consensus was reached among the survey participants regarding local infiltration (83%) and rapid growth of the lesion (81%) as markers of malignancy.

**Table 2:** Results from personal opinions

Markers of malignant adrenal lesion, *n* (%)	
Local infiltration	206 (83.1)
Rapid growth	201 (81.0)
Size > 6 cm	165 (66.5)
FDG-PET scan uptake	83 (33.5)
High radiodensity	45 (18.1)
Hormonal activity	36 (14.5)
History of cancer	34 (13.7)
Age > 65 years	18 (7.3)
Markers of difficult adrenalectomy, *n* (%)	
Radiological signs of local infiltration	180 (72.6)
Limited experience in MIS	173 (69.8)
Large lesion	148 (59.7)
Obesity	93 (37.5)
Not high-volume center	66 (26.6)
Bilateral lesions	20 (8.1)
Hormonal activity	18 (7.3)
Open surgery	13 (5.2)
Laparoscopic surgery	10 (4.0)
None	2 (0.8)
Small lesion	2 (0.8)
Robotic surgery	-
Most familiar approach, *n* (%)	
Lateral transperitoneal	125 (50.4)
Anterior transperitoneal	108 (43.5)
Lateral retroperitoneal	13 (5.2)
Prone retroperitoneal	2 (0.8)
Definition of large lesion, *n* (%)	
≥ 4 cm	39 (15.7)
≥ 5 cm	42 (16.9)
≥ 6 cm	137 (55.2)
≥ 8 cm	16 (6.5)
≥ 10 cm	14 (5.6)
Absolute contraindications to laparoscopic adrenalectomy, *n* (%)	
Limited experience in MIS	172 (69.4)
Radiological signs of local infiltration	105 (42.3)
Not high-volume center	73 (29.4)
Lesion size	70 (28.2)
None	33 (13.3)
High suspicion of malignancy, regardless of size	27 (10.9)
ASA score 3–4	17 (6.9)
Bilateral lesions	12 (4.8)
Obesity	10 (4.0)
Hormonal activity	5 (2.0)
Absolute contraindications to robotic adrenalectomy, *n* (%)	
Limited experience in MIS	160 (64.5)
Not high-volume center	87 (35.1)
Radiological signs of local infiltration	82 (33.1)
Lesion size	54 (21.8)
None	44 (17.7)
High suspicion of malignancy, regardless of size	20 (8.1)
Bilateral lesions	13 (5.2)
ASA 3–4	12 (4.8)
Obesity	8 (3.2)
Previous abdominal surgery	3 (1.2)
Hormonal activity	2 (0.8)
Surgeons who know the existence of an adrenal disease registry, *n* (%)	50 (20.2)
Surgeons who believe the development of a national adrenal disease registry is important, *n* (%)	236 (95.2)
Minimum number of adrenalectomies/year to define an expert surgeon, *n* (%)	
At least 20	61 (24.6)
21–40	90 (36.3)
41–100	70 (28.2)
> 100	7 (2.8)
Experience cannot be calculated based on the number of adrenalectomies alone	20 (8.1)
Minimum number of adrenalectomies/year to define a high-volume center, *n* (%)	
At least 20	103 (41.5)
At least 50	95 (38.3)
> 50	39 (15.7)
> 100	8 (3.2)
> 200	1 (0.4)
High-volume center cannot be calculated based on the number of adrenalectomies alone	2 (0.8)

*FDG-PET* fludeoxyglucose-18 positron emission tomography, *MIS* minimally invasive surgery, *ASA* American Society of Anesthesiologists

Lesion size > 6 cm emerged as important markers of malignancy too, but without reaching consensus (67%). Most participants believe that radiological signs of local infiltration (73%), limited surgeon experience (70%), and large lesion (60%) are the main predictive factors of difficult adrenalectomy. The lateral (125, 50%) and anterior transperitoneal (108, 44%) approaches were found to be the most familiar for Italian surgeons. Over half of the participants defined adrenal lesions measuring ≥ 6 cm as “large lesions”. Limited experience in MIS represents the main contraindication for both laparoscopic and robotic adrenalectomy. Radiological signs of local infiltration, not high-volume center and lesion size are other contraindications for MIS. While nearly all participants recognized the importance of a national adrenal disease registry, only 20% were aware of existing one. There was no clear consensus on defining either an expert surgeon or a high-volume center. However, 36% of participants believed that 20–40 adrenalectomies were required to be considered an expert surgeon, 42% believe that at least 20 adrenalectomies/year are necessary for a high-volume center, while 38% believe that at least 50 are necessary.

Table 3 reports results on the participants’ clinical practice section. The majority of the participants (221, 89%) perform < 10 adrenalectomies/year and practice at hospitals that perform < 20 adrenalectomies/ year (200, 81%). Almost half of the participating centers (122, 49%) have a multidisciplinary meeting on adrenal pathology, while approximately 30% have a dedicated adrenal surgery pathologist.

**Table 3:** Results from clinical practice

Number of adrenalectomies performed in your hospital on average in 1 year, *n* (%)	
0–10	126 (50.8)
11–20	74 (29.8)
21–30	29 (11.7)
31–40	11 (4.4)
> 40	8 (3.2)
Number of adrenalectomies performed by a single surgeon in a year, *n* (%)	
0–10	221 (89.1)
11–20	18 (7.3)
21–30	8 (3.2)
31–40	1 (0.4)
Median number of adrenalectomies performed in your hospital on average in 1 year, *n* (IQR 1–10)	
Open	1 (0–10)
Laparoscopic	8 (0–10)
Robotic	0 (0–10)
Cancer	3 (0–10)
Large lesions (> 6 cm)	4 (0–10)
Hospitals with a multidisciplinary meeting on adrenal pathology, *n* (%)	122 (49.2)
Hospitals with a pathologist dedicated to adrenal surgery, *n* (%)	76 (30.6)
Factors involved in the decision-making process for the surgical approach, *n* (%)	
Most familiar approach	157 (63.3)
Lesion size	52 (21.0)
Lesion side	16 (6.5)
Associated procedures	14 (5.6)
Hormonal activity	2 (0.8)
All the above	7 (2.8)
Surgical technique in malignant lesion (suspected or certain), *n* (%)	
Open	178 (71.8)
Robotic	36 (14.5)
Laparoscopic	34 (13.7)
Spleen fixing in left-sided adrenalectomy, *n* (%)	
Never	119 (48.0)
Spleen mobilization not required (submesocolic approach)	76 (30.6)
Glue	34 (13.7)
Stitch	11 (4.4)
Supine position for 24 h	8 (3.2)
Causes of conversion in case of MIS, *n* (%)	
Bleeding	181 (73)
Vascular infiltration	140 (56.5)
Technical difficulties (obesity, difficulty in finding the lesion)	84 (33.9)
Lesion size	51 (20.6)
Anesthetic reasons (hemodynamic instability)	22 (8.9)
Causes of re-intervention, *n* (%)	
Bleeding	207 (83.5)
Iatrogenic injury	125 (50.4)
Radicalization	50 (20.2)
Splenic infarction	23 (9.3)
Infection	10 (4.0)
Surgical drain use, yes (%): no (%)	176 (71)): 72 (29)
Median hospital stay, days (IQR)	3 (3–5)
Enhanced recovery items for adrenalectomy, *n* (%)	
Earlier mobilization	235 (94.8)
MIS	222 (89.5)
Individualized nutrition schedule	201 (81.0)
No antibiotic	180 (72.6)
No urinary catheter, or removal on postoperative day 1	142 (57.3)
Psychological and pain assessment	129 (52.0)
No/little perioperative intravenous fluid	124 (50.0)
No surgical drain or removal in case of < 30 ml	124 (50.0)
Oral functional solution until 2 h before operation	88 (35.5)
Drink upon awakening from anesthesia	56 (22.6)
Oral intake 6 h after surgery	52 (21.0)

*MIS* minimally invasive surgery, *IQR* interquartile range

The most familiar approach (157, 63%) and the lesion size (52, 21%) are the mainly involved factors in the decision-making process for the surgical approach. Hormonal activity (2, 1%), lesion side (16, 6.5%), and the need for associated procedures (14, 6%) appear to have a lesser influence on the choice of surgical approach. The open approach is the most frequently used in case of malignant lesion (178, 72%), although almost 30% of participants use MIS (laparoscopic or robotic) even in cases of suspicion or certainty of a malignant lesion.

During left-sided adrenalectomy, nearly half of the participants (119, 48%) do not fix the spleen, while spleen mobilization is not required in 31% of cases.

In case of MIS, bleeding emerged to be the most common cause of conversion to open surgery (181, 73%), followed by vascular infiltration (140, 57%), technical difficulties (84, 34%), and lesion size (51, 21%).

There was a consensus among the participants (207, 84%) regarding bleeding as the primary cause for re-intervention, followed by iatrogenic injuries (125, 50%) and the need of radicalization (50, 20%).

Regarding ERAS items for adrenalectomy, there is consensus on the early mobilization (235, 95%), the use of MIS (222, 90%), and individualized nutrition schedule (201, 81%). Almost 73% of the participants do not use antibiotic therapy; urinary catheter is not used or is removed on postoperative day one by 57% of the participants and surgical drain is placed by most of the participants (176, 71%).

## Discussion

The current study investigated the current state-of-the-art regarding adrenal surgery in Italy. We explored the risk of cancer in surgical adrenal lesions, the different surgical approaches for adrenalectomy and the perspectives of young and senior general surgeons on the definition of high-volume center and expert adrenal surgeon have been investigated.

To this aim, a 37-question survey was administered electronically to all SICE members and other eligible participants.

Overall, the responses of 248 surgeons were analyzed. Most of the participants were young (median age 36 years), men (172, 69%) and attending surgeons (201, 81%).

Consensus was reached among the survey participants regarding local infiltration (83%) and rapid growth of the lesion (81%) as markers of malignancy. Conversely, other independent risk factors for cancer reported in the literature (lesion size > 6 cm, high Hounsfield density, male sex, older age, non-incidental diagnosis and ^18^F-fluorodeoxyglucose positron emission tomography (PET) ratio > 1.5) did not appear to be as predictive of malignancy among Italian surgeons [12, 14, 23, 24].

Computed tomography (CT) is the most frequently employed imaging modality for adrenal evaluation. Magnetic resonance imaging (MRI) also plays a significant role in the assessment of adrenal pathology, particularly in delineating the extent of local invasion in cases of ACC [25].

In the absence of clear radiological signs of malignancy and no evidence of hormonal activity, there is no unanimous agreement regarding the appropriate radiological and biochemical follow-up, which should, therefore, be tailored to each individual patient [25, 26]. According to the American Association of Clinical Endocrinologists (AACE) and the American Association of Endocrine Surgeons (AAES), lesions < 4 cm without clear signs of malignancy undergo radiological evaluation at 3, 6, and 12 months [23]. American College of Radiology (ARC) suggests no follow-up for clearly benign lesions, 12-month follow-up for lesions up to 2 cm with dubious radiological signs, and 6-month follow-up for lesions > 2 cm. [27]. Finally, the ESE does not recommend radiological follow-up for benign lesions up to 4 cm and recommends evaluation at 6–12 months in other cases. Surgical resection is indicated in cases of > 20% lesion growth or the appearance of radiological signs of malignancy [28].

The literature indicates that the risk of adrenal cancer increases with lesion size, reaching almost 38% in lesions exceeding 6 cm, although this risk appears lower in more recent case studies [14, 22–24, 29]. However, size alone can not be a single tool for preoperative assessment of adrenal cancer. Since the risk of cancer is critical in choosing surgical approach [13, 14, 20], many several cancer risk stratification algorithms have been developed [30–33].

Based on the present Italian survey, only 15% of the participants considered hormonal activity a predictive factor for malignant adrenal lesion. Furthermore, hormonal activity does not represent a contraindication to laparoscopic and robotic adrenalectomy, nor a predictive factor for complex adrenalectomy for most of the participants.

The literature indicates that up to 60% of malignant adrenal lesions have hormonal activity, with hypercortisolism being a strong indicator of malignancy [12, 13].

Therefore, although many ACCs have hormonal activity and this correlates with a worse prognosis, hormonal activity is not considered a major predictive factor of malignant lesion, and this aligns our findings [12–14, 21, 22].

Although current guidelines suggest open adrenalectomy for malignant adrenal lesions [22, 34, 35, 38], several authors have reported the safety and feasibility of MIS also in these cases, not only in technical terms, but also in terms of oncological outcomes. However, most of the studies are retrospective and involved small samples sizes [29, 36, 37, 39].

Donatini et al*.* compared the outcome of laparoscopic to open adrenalectomy in patients affected by ACC smaller than 10 cm, reporting no differences in postoperative morbidity and long-term oncological outcome (at least 51 ± 43 months of median follow-up) between the two groups, with a significant shorter length of stay in case of MIS [34]. However, this retrospective cohort study included only 34 patients, which could potentially limit the statistical power of the analysis [38].

In this study, open adrenalectomy was considered the gold standard for suspected malignancy. However, almost 30% of the participants practice MIS even in case of malignancy and less than 10% considered the malignant tumor an absolute contraindication to MIS. These findings align with the literature, emphasizing the importance of R0 resection over the surgical approach [34, 35].

Guidelines emphasize the importance of solid experience in MIS and that limited experience in MIS represents the main absolute contraindication for both laparoscopic and robotic adrenalectomy [22, 34, 35], and these aspects are well known to the surgical Italian community, as confirmed by the results of this survey.

It is interesting to note that, according to our results, there is no unanimous agreement in Italy on the definition of expert surgeon and a high-volume center. Almost 40% of participants believed that 20–40 adrenalectomies were required to be considered an expert surgeon, while at least 20 procedures per year are required to define a high-volume center for almost 40% of participants. Furthermore, despite these results, most participants stated that patients affected by adrenal lesion are frequently treated by surgeons performing < 10 adrenalectomies per year in low-volume hospitals for adrenal surgery. These data reflect that an unanimous consensus is lacking worldwide [4, 34, 40, 41]. The European Society of Endocrinology (ESE)/European Network for the Study of Adrenal Tumors (ENSAT) clinical practice guidelines on the management of ACC, published in the 2018, recommend a minimum annual workload of 6 adrenalectomies per year, with a preference for more than 20 surgeries annually. Sufficient experience in oncological surgery is also crucial. For optimal clinical outcomes, the entire operative team, including anesthesiologists, should be well-trained in adrenal surgery [22].

In 2016, Palazzo et al*.,* and 1 year later, Anderson et al*.* reported increased complications, costs, and length of stay for adrenalectomies performed by low-volume surgeons (< 6 cases/year) [41, 42]. The authors’ starting point was precisely that, although studies on the safety of adrenalectomy come from high-volume centers, often, in clinical practice, the average surgeon who performs adrenalectomy handles only one case per year on average [4]. From this observation, arises the need to identify a threshold value of minimum adrenalectomies per surgeon to improve the surgical outcomes and centralize patients in high-volume centers [42].

In this survey, a multidisciplinary discussion is currently performed to validate surgical indications for adrenal lesions in approximately half of the hospitals (49%) and nearly one over three hospitals has a pathologist dedicated to adrenal surgery. Only 20% of participants are aware of the existence of an adrenal disease registry, but almost all (95%) agree on its importance.

Regarding the surgical technique aspect, since no clear superiority of one MIS approach over another (transperitoneal/retroperitoneal, anterior/lateral) in terms of perioperative outcomes has been demonstrated, the guidelines suggest that the surgeon should use the surgical approach for adrenalectomy with whom they are most familiar and the one that yields the best patient outcomes [33, 37].

As retrieved from the present survey, during the preoperative decision-making process, Italian surgeons choose the approach most familiar to them regardless of the patient’ or lesion’s characteristics. This results in the anterior and lateral transperitoneal approaches being selected by most of the participants.

Based on the findings of this survey and consistent with existing literature, other factors influencing the decision-making process include lesion size (21%), lesion site (6.5%), and the requirement for concomitant procedures (5.6%). As previously noted, lesion size correlates with the risk of malignancy, and some studies suggest superior outcomes with a transperitoneal approach compared to a retroperitoneal one [24, 28, 39, 43]. Regarding lesion laterality, as established in the literature, open right adrenalectomy is generally considered technically more challenging than the left one due to anatomical considerations (the right adrenal vein is shorter and drains directly into the inferior vena cava, in close proximity to the duodenum). Similarly, in MIS for left adrenalectomy, splenic flexure mobilization may result in an increased operative time compared to the open approach [44].

Although the retroperitoneal approach has been shown to be associated with better outcomes in cases of previous abdominal surgery or bilateral adrenalectomy, it is used by only 6% of participants in this survey [37].

There is considerable variability in surgical practice, with approximately one-third of the surveyed surgeons not performing spleen mobilization during left adrenalectomy. This variation may be attributed to the use of different surgical approaches, such as the retroperitoneal and the anterior transperitoneal submesocolic ones. This latter approach has proven to be feasible and safe even in the case of pheochromocytoma, allowing for early ligation of the adrenal vein, although it obviously requires extensive experience in MIS [45].

Bleeding was found to be the primary cause of conversion in case of MIS (73%) and for re-intervention (83.5%). Interestingly, more than 70% of survey participants reported using drain following adrenalectomy, even though the literature generally discourages routine drain for this procedure [46]. It should be noted that in the questions regarding the ERAS protocol, about half of the participants stated that they do not use drain or that they remove it if the output is < 30 ml/day; therefore, we believe that in the 70% who use it, a certain percentage removes it early. Another reason could be that, as emerges from the results of this survey, adrenalectomies are often performed by surgeons who have performed < 10 adrenalectomies per year in low-volume hospitals for adrenal surgery, this could determine a greater prudence on the surgeon’ part. Finally, it is widely recognized that drains are frequently used for prophylactic purposes and to provide mental reassurance to the surgeon.

Currently, little data are available in the literature regarding the application of ERAS pathway to adrenal surgery; however, ERAS protocols appear to be able to improve the perioperative outcomes of patients undergoing LA, in terms of pain control, hospital stay, and return to daily activities [47, 48]. According to our results, most of the participants report using early mobilization, MIS, personalized nutrition and do not use antibiotic prophylaxis.

In our opinion, both the disparity between the significance of surgical experience and the clinical practice of treating patients with adrenal lesion continue in low-volume centers, highlight the need for further studies with larger sample size.

The main limitation of the present study is that, although the sample is large (*n* = 248), there is a preponderance of young surgeons, which may limit the generalizability of the results to experienced surgeons, a common issue in survey-based research. Given that this study provides a contemporary snapshot of Italian adrenal surgery practice, rather than focusing on learning curves or specific surgical techniques, we considered the inclusion of residents appropriate. Finally, this paper represents the initial phase of an ongoing observational ambispective multicentric study registered with ClinicalTrials.gov (NCT03679468), which will report the clinical and surgical outcomes of a large patient cohort, ultimately enabling evidence-based conclusions. As a further limit, it focused on a single nation, which may yield divergent results in other countries. Moreover, expanding this inquiry to include urologists would be beneficial to explore potential variations in the treatment and management of patients presenting with adrenal lesions.

## Conclusion

In conclusion, based on this study, local infiltration and rapid growth emerged as the most significant markers of malignant adrenal lesions.

Open adrenalectomy remains the gold standard in cases of suspected malignancy, but almost 30% of the participants reported performing MIS even in case of malignancy and less than 10% considered the malignancy an absolute contraindication to MIS.

The lateral and anterior transperitoneal approaches were found to be the most commonly used techniques by Italian surgeons.

In Italy, patients affected by adrenal lesions are often treated by surgeons who perform < 10 adrenalectomies/year in low-volume hospitals. There is no unanimous consensus on the definition of expert surgeon and high-volume center and this is a critical open question. This study represents a starting point for an ongoing ambispective observational multicentric study.

## References

[1] Ginzberg SP, Gasior JA, Kelz LR, Passman JE, SoegaardBallester JM, Roses RE, Fraker DL, Wachtel H (2023) Adrenalectomy approach and outcomes according to surgeon volume. Am J Surg. 10.1016/j.amjsurg.2023.10.042 37940441 10.1016/j.amjsurg.2023.10.042 PMC10922122

[2] Thompson LH, Nordenström E, Almquist M, Jacobsson H, Bergenfelz A (2017) Risk factors for complications after adrenalectomy: results from a comprehensive national database. Langenbecks Arch Surg 402(2):315–322. 10.1007/s00423-016-1535-8 27896436 10.1007/s00423-016-1535-8 PMC5346413

[3] Kazaure HS, Sosa JA (2019) Volume-outcome relationship in adrenal surgery: a review of existing literature. Best Pract Res Clin Endocrinol Metabolism. 10.1016/j.beem.2019.101296 10.1016/j.beem.2019.101296 31331729

[4] Park HS, Roman SA, Sosa JA (2009) Outcomes from 3144 adrenalectomies in the United States: which matters more, surgeon volume or specialty? Arch Surg 144(11):1060–1067. 10.1001/archsurg.2009.191 19917944 10.1001/archsurg.2009.191

[5] Murphy MM, Witkowski ER, Ng SC, McDade TP, Hill JS, Larkin AC, Whalen GF, Litwin DE, Tseng JF (2010) Trends in adrenalectomy: a recent national review. Surg Endosc 24(10):2518–2526. 10.1007/s00464-010-0996-z 20336320 10.1007/s00464-010-0996-z

[6] Piccoli M, Pecchini F, Serra F, Nigro C, Colli G, Gozzo D, Zirilli L, Madeo B, Rochira V, Mullineris B (2021) Robotic versus laparoscopic adrenalectomy: pluriannual experience in a high-volume center evaluating indications and results. J Laparoendosc Adv Surg Tech A 31(4):375–381. 10.1089/lap.2020.0839 33450160 10.1089/lap.2020.0839 PMC8060876

[7] Sforza S, Minervini A, Tellini R, Ji C, Bergamini C, Giordano A, Lu Q, Chen W, Zhang F, Ji H, Di Maida F, Prosperi P, Masieri L, Carini M, Valeri A, Guo H (2021) Perioperative outcomes of robotic and laparoscopic adrenalectomy: a large international multicenter experience. Surg Endosc 35(4):1801–1807. 10.1007/s00464-020-07578-5 32328826 10.1007/s00464-020-07578-5

[8] Castillo OA, Vitagliano G, Secin FP, Kerkebe M, Arellano L (2008) Laparoscopic adrenalectomy for adrenal masses: does size matter? Urology 71(6):1138–1141. 10.1016/j.urology.2007.12.019 18336879 10.1016/j.urology.2007.12.019

[9] Young WF Jr (2000) Management approaches to adrenal incidentalomas. A view from Rochester, Minnesota. Endocrinol Metabolism Clin North America. 10.1016/s0889-8529(05)70122-5 10.1016/s0889-8529(05)70122-5 10732270

[10] Cawood TJ, Hunt PJ, O’Shea D, Cole D, Soule S (2009) Recommended evaluation of adrenal incidentalomas is costly, has high false-positive rates and confers a risk of fatal cancer that is similar to the risk of the adrenal lesion becoming malignant; time for a rethink? Eur J Endocrinol 161(4):513–527. 10.1530/EJE-09-0234 19439510 10.1530/EJE-09-0234

[11] Balla A, Corallino D, Ortenzi M, Palmieri L, Meoli F, Guerrieri M, Paganini AM (2022) Cancer risk in adrenalectomy: are adrenal lesions equal or more than 4 cm a contraindication for laparoscopy? Surg Endosc 36(2):1131–1142. 10.1007/s00464-021-08380-7 33650006 10.1007/s00464-021-08380-7 PMC8758647

[12] Kahramangil B, Kose E, Remer EM, Reynolds JP, Stein R, Rini B, Siperstein A, Berber E (2022) A modern assessment of cancer risk in adrenal incidentalomas: analysis of 2219 patients. Ann Surg. 10.1097/SLA.0000000000004048 32541223 10.1097/SLA.0000000000004048

[13] Bilimoria KY, Shen WT, Elaraj D, Bentrem DJ, Winchester DJ, Kebebew E, Sturgeon C (2008) Adrenocortical carcinoma in the United States: treatment utilization and prognostic factors. Cancer. 10.1002/cncr.23886 18973179 10.1002/cncr.23886

[14] Vanbrabant T, Fassnacht M, Assie G, Dekkers OM (2018) Influence of hormonal functional status on survival in adrenocortical carcinoma: systematic review and meta-analysis. Eur J Endocrinol 179(6):429–436. 10.1530/EJE-18-0450 30325179 10.1530/EJE-18-0450

[15] Iñiguez-Ariza NM, Kohlenberg JD, Delivanis DA, Hartman RP, Dean DS, Thomas MA, Shah MZ, Herndon J, McKenzie TJ, Arlt W, Young WF Jr, Bancos I (2017) Clinical, biochemical, and radiological characteristics of a single-center retrospective cohort of 705 large adrenal tumors. Mayo Clinic Proc Innov Quality Outcomes 2(1):30–39. 10.1016/j.mayocpiqo.2017.11.002 10.1016/j.mayocpiqo.2017.11.002 PMC6124341 30225430

[16] Vural V, Kılınç EM, Sarıdemir D, Gök İB, Hüseynov A, Akbarov A, Yaprak M (2020) Association between tumor size and malignancy risk in hormonally inactive adrenal incidentalomas. Cureus 12(1):e6574. 10.7759/cureus.6574 32051792 10.7759/cureus.6574 PMC7001135

[17] Bergamini C, Martellucci J, Tozzi F, Valeri A (2011) Complications in laparoscopic adrenalectomy: the value of experience. Surg Endosc 25(12):3845–3851 21681621 10.1007/s00464-011-1804-0

[18] Code of Conduct for responsible Research. World Health Organization (WHO). 2017. https://www.who.int/about/ethics/code-of-conduct-responsible-research.pdf.

[19] Eysenbach G. (2004). Improving the quality of Web surveys: the Checklist for Reporting Results of Internet E-Surveys (CHERRIES). J Med Internet Res. Sep 29;6(3):e34. 10.2196/jmir.6.3.e34. Erratum in: 10.2196/jmir.2042. 10.2196/jmir.6.3.e34 PMC1550605 15471760

[20] Diamond IR, Grant RC, Feldman BM, Pencharz PB, Ling SC, Moore AM, Wales PW (2014) Defining consensus: a systematic review recommends methodologic criteria for reporting of Delphi studies. J Clin Epidemiol 67(4):401–409. 10.1016/j.jclinepi.2013.12.002. (**PMID: 24581294**) 24581294 10.1016/j.jclinepi.2013.12.002

[21] Fassnacht M, Dekkers OM, Else T, Baudin E, Berruti A, de Krijger R, Haak HR, Mihai R, Assie G, Terzolo M (2018) European Society of Endocrinology Clinical Practice Guidelines on the management of adrenocortical carcinoma in adults, in collaboration with the European Network for the Study of Adrenal Tumors. Eur J Endocrinol 179(4):G1–G46. 10.1530/EJE-18-0608 30299884 10.1530/EJE-18-0608

[22] Cyranska-Chyrek E, Szczepanek-Parulska E, Olejarz M, Ruchala M (2019) Malignancy risk and hormonal activity of adrenal incidentalomas in a large cohort of patients from a single tertiary Reference center. Int J Environ Res Public Health 16(10):1872. 10.3390/ijerph16101872 31137898 10.3390/ijerph16101872 PMC6571894

[23] Zeiger MA, Thompson GB, Duh QY, Hamrahian AH, Angelos P, Elaraj D, Fishman E, Kharlip J, Association A, of Clinical Endocrinologists, American Association of Endocrine Surgeons, (2009) American Association of clinical endocrinologists and American Association of endocrine surgeons medical guidelines for the management of adrenal incidentalomas: executive summary of recommendations. Endocr Pract 15(5):450–453. 10.4158/EP.15.5.450 19632968 10.4158/EP.15.5.450

[24] Balla A, Palmieri L, Meoli F, Corallino D, Ortenzi M, Ursi P, Guerrieri M, Quaresima S, Paganini AM (2020) Are adrenal lesions of 6 cm or more in diameter a contraindication to laparoscopic adrenalectomy? A case-control study. A case-control study World J Surg 44(3):810–818. 10.1007/s00268-019-05287 31728629 10.1007/s00268-019-05287-2

[25] Jason DS, Oltmann SC (2019) Evaluation of an Adrenal Incidentaloma. The Surgical clinics of North America 99(4):721–729. 10.1016/j.suc.2019.04.009 31255202 10.1016/j.suc.2019.04.009

[26] Terzolo M, Stigliano A, Chiodini I, Loli P, Furlani L, Arnaldi G, Reimondo G, Pia A, Toscano V, Zini M, Borretta G, Papini E, Garofalo P, Allolio B, Dupas B, Mantero F, Tabarin A, Association I, of Clinical Endocrinologists, (2011) AME position statement on adrenal incidentaloma. Eur J Endocrinol 164(6):851–870. 10.1530/EJE-10-1147 21471169 10.1530/EJE-10-1147

[27] Mayo-Smith WW, Song JH, Boland GL, Francis IR, Israel GM, Mazzaglia PJ, Berland LL, Pandharipande PV (2017) Management of Incidental Adrenal Masses: A White Paper of the ACR Incidental Findings Committee. Journal of the American College of Radiology: JACR 14(8):1038–1044. 10.1016/j.jacr.2017.05.001 28651988 10.1016/j.jacr.2017.05.001

[28] Fassnacht M, Arlt W, Bancos I, Dralle H, Newell-Price J, Sahdev A, Tabarin A, Terzolo M, Tsagarakis S, Dekkers OM (2016) Management of adrenal incidentalomas: European Society of Endocrinology Clinical Practice Guideline in collaboration with the European Network for the Study of Adrenal Tumors. Eur J Endocrinol 175(2):G1–G34. 10.1530/EJE-16-0467 27390021 10.1530/EJE-16-0467

[29] NIH (2002) NIH state-of-the-science statement on management of the clinically inapparent adrenal mass (“incidentaloma”). NIH Consens State Sci Statements 19(2):1–25 14768652

[30] Birsen O, Akyuz M, Dural C, Aksoy E, Aliyev S, Mitchell J, Siperstein A, Berber E (2014) A new risk stratification algorithm for the management of patients with adrenal incidentalomas. Surgery 156(4):959–965. https ://doi.org/10.1016/j.surg.2014.06.042 10.1016/j.surg.2014.06.042 25239353

[31] Amodru V, Taieb D, Guerin C, Paladino NC, Brue T, Sebag F, Castinetti F (2019) Large adrenal incidentalomas require a dedicated diagnostic procedure. Endocr Pract 25(7):669–677.https ://doi.org/10.4158/EP-2018-0616 10.4158/EP-2018-0616 30865539

[32] Alberici L, Paganini AM, Ricci C, Balla A, Ballarini Z, Ortenzi M, Casole G, Quaresima S, Di Dalmazi G, Ursi P, Alfano MS, Selva S, Casadei R, Ingaldi C, Lezoche G, Guerrieri M, Minni F, Tiberio GAM (2022) Development and validation of a preoperative “difficulty score” for laparoscopic transabdominal adrenalectomy: a multicenter retrospective study. Surg Endosc 36(5):3549–3557. 10.1007/s00464-021-08678-6 34402981 10.1007/s00464-021-08678-6 PMC9001553

[33] Fassnacht M, Assie G, Baudin E, Eisenhofer G, de la Fouchardiere C, Haak HR, de Krijger R, Porpiglia F, Terzolo M, Berruti A, ESMO Guidelines Committee (2020) Adrenocortical carcinomas and malignant phaeochromocytomas: ESMO-EURACAN clinical practice guidelines for diagnosis, treatment and follow-up. Ann Oncol 31(11):1476–1490 32861807 10.1016/j.annonc.2020.08.2099

[34] Gaujoux S, Mihai R, Joint working group of ESES and ENSAT (2017) European Society of Endocrine Surgeons (ESES) and European Network for the Study of Adrenal Tumours (ENSAT) recommendations for the surgical management of adrenocortical carcinoma. Br J Surg 104(4):358–376. 10.1002/bjs.10414 28199015 10.1002/bjs.10414

[35] Corallino D, Balla A, Palmieri L, Sperduti I, Ortenzi M, Guerrieri M, Paganini AM (2023) Is transperitoneal laparoscopic adrenalectomy for pheochromocytoma really more challenging? A propensity score-matched analysis. J Endocrinol Invest 46(8):1589–1596. 10.1007/s40618-023-02013-7 36705839 10.1007/s40618-023-02013-7 PMC10348962

[36] Arolfo S, Giraudo G, Franco C, Parasiliti Caprino M, Seno E, Morino M (2022) Minimally invasive adrenalectomy for large pheochromocytoma: not recommendable yet? Results from a single institution case series. Langenbecks Arch Surg 407(1):277–283. 10.1007/s00423-021-02312-8 34468864 10.1007/s00423-021-02312-8 PMC8847286

[37] Stefanidis D, Goldfarb M, Kercher KW et al (2013) SAGES guidelines for minimally invasive treatment of adrenal pathology. Surg Endosc 27(11):3960–3980. 10.1007/s00464-013-3169-z 24018761 10.1007/s00464-013-3169-z

[38] Donatini G, Caiazzo R, Do Cao C, Aubert S, Zerrweck C, El-Kathib Z, Gauthier T, Leteurtre E, Wemeau JL, Vantyghem MC, Carnaille B, Pattou F (2014) Long-term survival after adrenalectomy for stage I/II adrenocortical carcinoma (ACC): a retrospective comparative cohort study of laparoscopic versus open approach. Ann Surg Oncol 21(1):284–291. 10.1245/s10434-013-3164-6 24046101 10.1245/s10434-013-3164-6

[39] Kerkhofs TM, Verhoeven RH, Bonjer HJ, van Dijkum EJ, Vriens MR, De Vries J, Van Eijck CH, Bonsing BA, Van de Poll-Franse LV, Haak HR, Network DA (2013) Surgery for adrenocortical carcinoma in The Netherlands: analysis of the national cancer registry data. Eur J Endocrinol 169(1):83–89. 10.1530/EJE-13-0142 23641018 10.1530/EJE-13-0142

[40] Lombardi CP, Raffaelli M, Boniardi M, De Toma G, Marzano LA, Miccoli P, Minni F, Morino M, Pelizzo MR, Pietrabissa A, Renda A, Valeri A, De Crea C, Bellantone R (2012) Adrenocortical carcinoma: effect of hospital volume on patient outcome. Langenbecks Arch Surg 397(2):201–207. 10.1007/s00423-011-0866-8 22069043 10.1007/s00423-011-0866-8

[41] Palazzo F, Dickinson A, Phillips B, Sahdev A, Bliss R, Rasheed A, Krukowski Z, Newell-Price J (2016) Adrenal surgery in England: better outcomes in high-volume practices. Clin Endocrinol 85(1):17–20. 10.1111/cen.13021 10.1111/cen.13021 26776382

[42] Anderson KL Jr, Thomas SM, Adam MA, Pontius LN, Stang MT, Scheri RP, Roman SA, Sosa JA (2018) Each procedure matters: threshold for surgeon volume to minimize complications and decrease cost associated with adrenalectomy. Surgery 163(1):157–164. 10.1016/j.surg.2017.04.028 29122321 10.1016/j.surg.2017.04.028

[43] Nigri G, Rosman AS, Petrucciani N, Fancellu A, Pisano M, Zorcolo L, Ramacciato G, Melis M (2013) Meta-analysis of trials comparing laparoscopic transperitoneal and retroperitoneal adrenalectomy. Surgery 153(1):111–119. 10.1016/j.surg.2012.05.042 22939744 10.1016/j.surg.2012.05.042

[44] Kokorak L, Soltes M, Vladovic P, Marko L (2016) Laparoscopic left and right adrenalectomy from an anterior approach - is there any difference? Outcomes in 176 consecutive patients. Wideochirurgia i inne techniki maloinwazyjne. Videosurg Other Miniinvasive Techniques. 11(4):268–273 10.5114/wiitm.2016.64767 PMC5299086 28194247

[45] Balla A, Quaresima S, Ortenzi M, Palmieri L, Meoli F, Corallino D, Guerrieri M, Ursi P, Paganini AM (2019) Results after laparoscopic left anterior transperitoneal submesocolic adrenalectomy for the treatment of pheochromocytoma. Ann Ital Chir 90:220–224 31354147

[46] Chai S, Pan Q, Liang C, Zhang H, Xiao X, Li B (2021) Should surgical drainage after lateral transperitoneal laparoscopic adrenalectomy be routine?-A retrospective comparative study. Gland Surg 10(6):1910–1919. 10.21037/gs-20-829 34268075 10.21037/gs-20-829 PMC8258894

[47] Lelli G, Micalizzi A, Iossa A, Fassari A, Concistre A, Circosta F, Petramala L, De Angelis F, Letizia C, Cavallaro G (2024) Application of enhanced recovery after surgery (ERAS) protocols in adrenal surgery: a retrospective, preliminary analysis. J Minimal Access Surg 20(2):163–168. 10.4103/jmas.jmas_319_22 10.4103/jmas.jmas_319_22 PMC11095811 37282440

[48] He Z, Chen S, Lu M, Luo Y, Liu T, Xiao Y, Wang X (2022) A combination of laparoscopic approach and ERAS pathway optimizes outcomes and cost for adrenalectomy. Updat Surg 74(2):519–525. 10.1007/s13304-021-01188-z 10.1007/s13304-021-01188-z 34635985

