# Correction: Current state-of-the-art of adrenal surgery in Italy: the cancer risk in surgical adrenal lesions (CRISAL) survey

**DOI:** 10.1007/s13304-025-02306-x

**Published:** 2025-06-26

**Authors:** Diletta Corallino, Roberto Passera, Marco Inama, CRISAL (Cancer Risk In Surgical Adrenal Lesions) Collaborative Group, Diletta Corallino, Marco Inama, Francesca Abbatini, Stefano Agnesi, Ferdinando Agresta, Alberto Aiolfi, Laura Alberici, Giovanni Alemanno, Marco Ettore Allaix, Michele Ammendola, Pietro Maria Amodio, Marco Anania, Andrea Pisani Ceretti, Jacopo Andreuccetti, Roberta Angelico, Pierluigi Angelini, Mario Annecchiarico, Alfredo Annicchiarico, Pietro Anoldo, Amedeo Antonelli, Massimiliano Ardu, Giulio Argenio, Gabriela Aracelly Arroyo Murillo, Riccardo Avantifiori, Giulia Bagaglini, Gian Luca Baiocchi, Edoardo Baldini, Alberto Balduzzi, Francesco Balestra, Andrea Balla, Filippo Banchini, Elisa Bannone, Ilaria Benzoni, Lorenza Beomonte Zobel, Francesco Bianco, Arianna Birindelli, Cristina Bombardini, Luca Domenico Bonomo, Andrea Bottari, Marta Botti, Paolo Brazzarola, Francesco Brucchi, Simone Buccianti, Oreste Claudio Buonomo, Giacomo Calini, Roberto Cammarata, Tommaso Campagnaro, Sonia Cappelli, Marianna Capuano, Filippo Carannante, Gabriele Carbone, Luca Cardinali, Francesco Maria Carrano, Gianmaria Casoni Pattacini, Gianluca Cassese, Elisa Cassinotti, Antonio Castaldi, Fausto Catena, Giuseppe Cavallaro, Graziano Ceccarelli, Marta Celiento, Giovanni Cestaro, Vittorio Cherchi, Pasquale Cianci, Bruno Cirillo, Marco Clementi, Lucrezia Clocchiatti, Diego Coletta, Annalisa Comandatore, Luigi Eduardo Conte, Giovanni Conzo, Alessandro Coppola, Maurizio Costantini, Mihail Creciun, Diego Cuccurullo, Giuseppe Currò, Anna D’Amore, Maria Vittoria D’Addetta, Giorgio Dalmonte, Michele De Capua, Giuseppe Massimiliano De Luca, Maurizio De Luca, Nicolò De Manzini, Paolino De Marco, Belinda De Simone, Federico De Stefano, Sara Dedoni, Daniele Delogu, Annamaria De Bella, Giuseppe De Buono, Armando De Dato, Giacomo Di Filippo, Gregorio Di Franco, Nicola Di Lorenzo, Salomone Di Saverio, Andrea Divizia, Stefano D’Ugo, Ugo Elmore, Kevin Episodio, Emilio Eugeni, Giuseppe Evola, Nicolò Falco, Chiara Fantozzi, Alessia Fassari, Salvatore Fazzotta, Agostino Fernicola, Federico Festa, Irene Fiume, Tommaso Fontana, Edoardo Forcignanó, Gianluca Fornoni, Laura Fortuna, Alice Francescato, Marzia Franceschilli, Pietro Fransvea, Francesco Frattini, Giuseppe Frazzetta, Niccolò Furbetta, Raffaele Galleano, Giovanni Maria Garbarino, Enza Gelormini, Omar Ghazouani, Marco Giacometti, Alessio Giordano, Francesco Giovanardi, Giuseppe Giuliani, Ugo Giustizieri, Simone Guadagni, Tommaso Guagni, Anna Guariniello, Andrea Martina Guida, Giulio Iacob, Salvatore Incardona, Sara Ingallinella, Zoe Larghi Laureiro, Sara Lauricella, Leandro Siragusa, Silvana Leanza, Luca Lepre, Enrico Lodo, Sara Lucchese, Andrea Lucchi, Luigi Luzza, Andrea Pierre Luzzi, Carmen Maccagnano, Federico Maggi, Tommaso Maria Manzia, Sara Maritato, Nirvana Maroni, Riccardo Marsengo, Irene Marziali, Manuela Mastronardi, Marco Materazzo, Angela Maurizi, Gennaro Mazzarella, Francesca Meoli, David Merlini, Ilenia Merlini, Alessandra Micalizzi, Michail Vailas, Michele Minuto, Sarah Molfino, Serena Molica, Luca Morelli, Andrea Morini, Barbara Mullineris, Bruno Nardo, Giuseppe Navarra, Antonella Nicotera, Greta Olivari, Stefano Olmi, Monica Ortenzi, Paolo Ossola, Luca Ottaviani, Mario Pacilli, Alessandro M. Paganini, Livia Palmieri, Giuseppe Palomba, Vincenzo Papagni, Giulia Paradiso, Rocco Pasqua, Federico Passagnoli, Francesco Pata, Alberto Patriti, Giovanna Pavone, Domiziana Pedini, Fabio Pelle, Marco Pellicciaro, Vito Pende, Francesco Pennestrì, Bruno Perotti, Teresa Perra, Nicola Perrotta, Filippo Petrelli, Niccolò Petrucciani, Biagi Picardi, Andrea Picchetto, Stefania Piccioni, Chiara Piceni, Giulia Pietricola, Felice Pirozzi, Paolo Pizzini, Mauro Podda, Gaetano Poillucci, Alberto Porcu, Gianmario Edoardo Porcu, Priscilla Francesca Procopio, Lorenzo Provinciali, Francesco Puccetti, Ilaria Puccica, Eleonora Rapanotti, Antonia Rizzuto, Fabrizio Romano, Riccardo Rosati, Francesco Roscio, Leonardo Rossi, Stefano Rossi, Margherita Sandano, Federica Saraceno, Alberto Sartori, Paolina Saullo, Giovanni Scudo, Ardit Seitaj, Bruno Sensi, Marta Spalluto, Domenico Tamburrino, Mariarita Tarallo, Ernesto Tartaglia, Nicola Tartaglia, Giovanni Terrosu, Pier Luigi Tilocca, Flavio Tirelli, Luca Tirloni, Lorenza Trentavizi, Sofia Usai, Valeria Usai, Alessandro Ussia, Samuele Vaccari, Maria Rosaria Valenti, Gianluca Vanni, Samantha Vellei, Paolo Vincenzi, Antonio Vitiello, Mattia Zambon, Daniele Zigiotto, Maurizio Zizzo, Ugo Boggi, Riccardo Casadei, Massimiliano Fabozzi, Mario Guerrieri, Gabriele Materazzi, Gianluigi Moretto, Micaela Piccoli, Paolo Prosperi, Chiara Dobrinja, SICE (Italian Society of Endoscopic Surgery and New Technologies)

**Affiliations:** 1 Hepatobiliary Surgery Division, IRCCS San Raffaele Scientific Institute, Via Olgettina 60, 20132 Milan, Italy; 2 Department of General Surgery and Surgical Specialties, Sapienza University of Rome, Viale del Policlinico 155, 00161 Rome, Italy; 3 Nuclear Medicine, Department of Medical Sciences, AOU Città Della Salute E Della Scienza Di Torino, University of Turin, 10126 Turin, Italy; 4 General and Mininvasive Surgery Department, Pederzoli Hospital, Peschiera del Garda, Verona, Italy

**Correction: Updates in Surgery** 10.1007/s13304-025-02139-8

In this article some authors name were missing from the CRISAL collaborative group. These authors are list.

• Ugo Boggi

• Riccardo Casadei

• Massimiliano Fabozzi

• Mario Guerrieri

• Gabriele Materazzi

• Gianluigi Moretto

• Micaela Piccoli

• Paolo Prosperi

• Chiara Dobrinja

The original article has been corrected.

